# Polynucleotides in Aesthetic Medicine: A Review of Current Practices and Perceived Effectiveness

**DOI:** 10.3390/ijms25158224

**Published:** 2024-07-27

**Authors:** Kar Wai Alvin Lee, Kwin Wah Lisa Chan, Angela Lee, Cheuk Hung Lee, Jovian Wan, Sky Wong, Kyu-Ho Yi

**Affiliations:** 1 EverKeen Medical Centre, Hong Kong; alvin429@yahoo.com (K.W.A.L.); drchan.everkeen@gmail.com (K.W.L.C.); andylee618@hotmail.com (C.H.L.); 2 The Skin Oracle, Hong Kong; ang3la.l33@gmail.com; 3 Asia-Pacific Aesthetic Academy, Hong Kong; jovian.wan@apaa.org; 4 Leciel Medical Centre, Hong Kong; drskywong@gmail.com; 5 Division in Anatomy and Developmental Biology, Department of Oral Biology, Human Identification Research Institute, BK21 FOUR Project, Yonsei University College of Dentistry, 50-1 Yonsei-ro, Seodaemun-gu, Seoul 03722, Republic of Korea; 6 Maylin Clinic (Apgujeong), Seoul, Republic of Korea

**Keywords:** polynucleotides, cosmetic medicine, aesthetic medicine, skin texture, wrinkle depth, facial appearance

## Abstract

Polynucleotides, complex molecules composed of nucleotides, have gained attention in aesthetic medicine for their potential to regulate gene expression and promote tissue regeneration. This review aims to provide an overview of the current practices and perceived effectiveness of polynucleotides in aesthetic medicine. A comprehensive search of the literature was conducted using keywords related to polynucleotides, cosmetic application, and aesthetic application. Studies were selected based on their relevance to aesthetic medicine and the inclusion of human subjects. The review found that polynucleotides have been used to improve skin texture, reduce wrinkle depth, and enhance facial appearance. The studies reported varying degrees of efficacy and safety, with some studies demonstrating significant improvements in skin elasticity and hydration. However, others reported limited or no benefits. The review also highlighted the need for further research to establish the optimal use and efficacy of polynucleotides in aesthetic medicine. While the existing literature suggests that polynucleotides may have potential benefits in aesthetic medicine, more research is needed to fully understand their mechanisms of action and optimal use. Clinicians should be aware of the current limitations and potential risks associated with the use of polynucleotides in aesthetic medicine.

## 1. Introduction

The quest for a youthful and radiant appearance has driven the development of various aesthetic treatments, with a growing emphasis on harnessing naturally derived compounds in aesthetic medicine. Among these, polynucleotides (PNs) have gained significant attention due to their potential to regulate gene expression and promote tissue regeneration. PN are complex molecules composed of nucleotides, which are the building blocks of DNA and RNA. They have been shown to play a crucial role in various cellular processes, including cell proliferation, differentiation, and survival [1,2,3,4,5].

In the context of aesthetic medicine, polynucleotides have been investigated for their potential to improve skin texture, reduce wrinkle depth, and enhance facial appearance. The use of polynucleotides has been reported to stimulate collagen production, improve skin elasticity, and reduce inflammation [6]. Additionally, they have been shown to promote hair growth [7] and improve the appearance of scars [8].

Despite the growing interest in polynucleotides for aesthetic purposes, there is a need for a comprehensive review of the existing literature to evaluate their efficacy and optimal use in different indications. This review aims to provide an overview of the current practices and perceived effectiveness of polynucleotides in aesthetic medicine, with a focus on their use in facial rejuvenation, acne scars, and other aesthetic conditions. The findings of this review will provide clinicians with valuable insights into the potential benefits and limitations of polynucleotides in aesthetic medicine, enabling them to make informed decisions about their use in clinical practice.

Polydeoxyribonucleotide (PDRN) is emerging as a highly promising biomaterial in the fields of regenerative medicine and dermatology. Derived from salmon sperm, PDRN is composed of a mixture of deoxyribonucleotides that play a crucial role in cellular regeneration and tissue repair. Its multifaceted biological activities, including the stimulation of cell proliferation, angiogenesis, and anti-inflammatory effects, make PDRN an attractive candidate for a wide range of therapeutic applications. They are known to be explained by two pathways [9,10,11,12,13,14,15,16,17,18,19].

In the A2 receptor stimulation pathway, the process begins with the activation of the A2 receptor by a ligand, which subsequently activates the Gs protein. This activation stimulates adenylate cyclase (Ac), leading to the production of cyclic AMP (cAMP). The increase in cAMP activates protein kinase A (Pka), which then activates various transcription factors such as NFκB, CREB, and HIF-1. These transcription factors are crucial for cellular responses to hypoxia, inflammation, and other stimuli. The pathway’s final effects are on vascular endothelial growth factor (Vegf) and angiopoietin, which are critical for angiogenesis—the formation of new blood vessels. This process enhances blood flow and oxygen delivery to tissues, promoting healing and regeneration [2,4,20,21,22,23,24,25].

**Figure 1:**
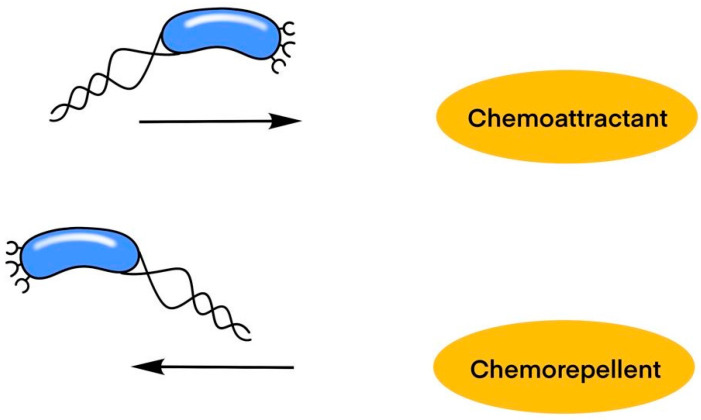
The higher concentration of DNA particles enhances environmental conditions, leading to increased cellular proliferation and regeneration. The more stressful environmental conditions, the less cellular proliferation there would be.

The salvage pathway, depicted on the right, involves nucleotide synthesis, which is essential for recycling nucleotides from degraded DNA. DNA synthesis happens where purine and pyrimidine bases are adequate. DNA nucleases (DNase) break down DNA into its component nucleotides, which can then be salvaged and reincorporated into new DNA molecules. These bases—purines (adenine and guanine) and pyrimidines (cytosine and thymine)—are critical building blocks for DNA synthesis, ensuring the cell maintains a sufficient supply of nucleotides for replication and repair (Figure 1).

**Figure 2:**
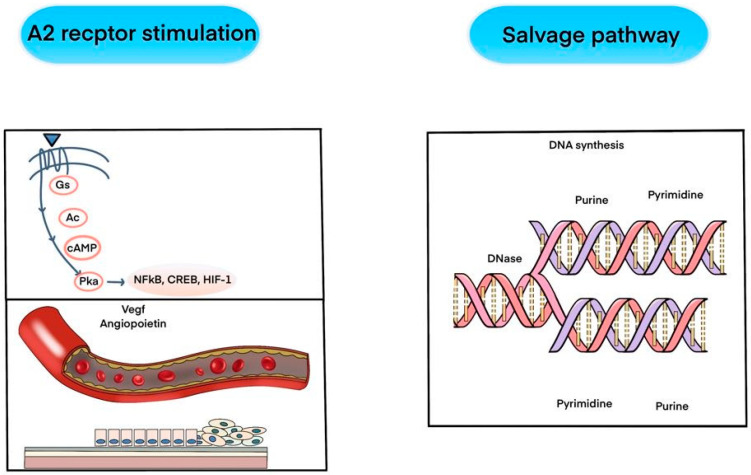
Polydeoxynucleotide (PDRN) has been used in two ways: adenosine A2A receptor stimulation and salvage pathway. And in many cases, we explain the polynucleotide in the same pathway, but no research has yet revealed the mechanism. The image depicts two pathways: A2 receptor stimulation, which activates a cascade leading to angiogenesis via VEGF and angiopoietin, and the salvage pathway, which recycles nucleotides for DNA synthesis by breaking down and reusing purine and pyrimidine bases.

PN is currently explained through the mechanisms of polydeoxyribonucleotide (PDRN), highlighting its role in cellular regeneration, tissue repair, and its various biological activities, such as stimulating cell proliferation, promoting angiogenesis, and exhibiting anti-inflammatory effects (Figure 2).

Keywords including “polynucleotide”, “cosmetic application”, “aesthetic application”, “clinical application”, “aesthetic use”, “aesthetic treatment” were searched in the MEDLINE, PubMed, and Ovid databases for relevant studies published on clinical trials, diagnosis, and treatment. Some papers were further reviewed using a double-blinding approach, sample size, control usage, randomization usage, and objective endpoint measurements. The studies in this review were classified according to the Oxford Center for Evidence-Based Medicine (OCEBM) evidence hierarchy: Level I includes systematic reviews or meta-analyses of randomized controlled trials (RCTs) or high-quality RCTs with low risk of bias. Level II consists of individual RCTs or systematic reviews of cohort studies with strong methodology. Level III covers cohort studies, case-control studies with less rigorous methodology, or systematic reviews of case series. Level IV includes case series and poor-quality cohort and case-control studies. Level V comprises expert opinion, case reports, or evidence based on physiology, bench research, or first principles.

## 2. Polynucleotide for General Skin Condition

Polynucleotides (PNs) in aesthetic medicine, as evidenced by various studies, demonstrate significant potential for improving skin conditions by stimulating collagen production, enhancing skin elasticity and hydration, and reducing fine lines and wrinkles, with applications ranging from skin boosters and cosmeceuticals to fillers and biorevitalization protocols (Table 1).

Yi et al. [26] provide an overview of skin boosters, which are injectable treatments used to improve skin texture and appearance. The authors focus on the role of polynucleotides, specifically PN, as a key component of skin boosters. The article defines skin boosters as a type of injectable treatment that stimulates collagen production, improves skin elasticity, and enhances skin hydration. The authors discuss the various classifications of skin boosters, including those containing PN, hyaluronic acid, and hyaluronic acid-hydrolysate. The authors highlight the benefits of PN-based skin boosters, including their ability to stimulate collagen synthesis, improve skin elasticity, and enhance tissue hydration. They note that PN has been shown to have anti-inflammatory and antioxidant properties, making it an attractive ingredient for skin rejuvenation treatments. The article also discusses the mechanisms of action of PN in skin boosters, including its ability to stimulate fibroblasts and keratinocytes to produce collagen and hyaluronic acid. The authors suggest that PN-based skin boosters may be particularly effective in improving skin texture, reducing fine lines and wrinkles, and enhancing facial rejuvenation (Level V).

Morganti et al. [27] explore the use of PN as a novel delivery system for cosmeceuticals. The authors discuss the potential of PN, a non-woven tissue composed of biodegradable fibers, to improve the bioavailability and stability of aesthetic ingredients. The authors highlight the unique properties of PN, including its biodegradability, biocompatibility, and ability to release active ingredients in a controlled manner. They suggest that PN can be used to deliver a range of aesthetic ingredients, including moisturizers, antioxidants, and vitamins, with potential applications in skin care products. The authors also discuss the advantages of using PN as a carrier system, including its ability to improve skin penetration and bioavailability, reduce irritation and allergic reactions, and provide sustained release of active ingredients (Level IIa).

Yi et al. [28] review the various types of skin boosters used in aesthetic treatments. The authors focus on the application of PN as a skin booster. PNs are biodegradable polymers that can stimulate collagen production, improve skin elasticity, and reduce the appearance of fine lines and wrinkles. The authors discuss the mechanism of action of PN, which involves the release of growth factors that stimulate collagen synthesis and improve skin texture. The authors review several studies that have used PN as a skin booster, including a randomized controlled trial that found significant improvements in skin elasticity and wrinkles after treatment with PN. They also discuss the potential benefits of combining PN with other skin boosters, such as hyaluronic acid and poly-L-lactic acid (Level Ia).

Lis et al. [29] investigate the use of PN as a treatment for reducing signs of aging in the neck skin. The authors conducted a case study to compare the efficacy of PN injections with different dosing intervals. The authors found that PN injections significantly improved skin elasticity, reduced wrinkles, and enhanced skin firmness in patients with neck skin aging. The study compared two different dosing regimens: a single injection session with a high dose of PN, and multiple injections with a lower dose spaced out over time. The results showed that both regimens improved skin appearance, but the multiple injection regimen produced more sustained results (Level IV).

Cesare et al. [30] describe a combined treatment protocol for biorevitalization that includes the use of PN as one of its components. The authors aim to provide a comprehensive approach to facial rejuvenation by combining PN with other treatments, including hyaluronic acid, growth factors, and platelet-rich plasma. The PN used in the protocol is composed of deoxyribonucleic acid (DNA) and ribonucleic acid (RNA), which are derived from animal sources. The authors suggest that these PNs can stimulate collagen synthesis, improve skin elasticity, and reduce wrinkles. The authors report a case series of 20 patients who underwent the combined treatment protocol, which included injections of PN, hyaluronic acid, and growth factors. The results showed significant improvements in skin elasticity, firmness, and wrinkles, as well as an increase in collagen synthesis (Level IV).

Samizadeh et al. [35] provide an overview of the role of PN in aesthetic medicine. PN are complex biomolecules composed of DNA and RNA that have been found to play a crucial role in various biological processes, including wound healing, tissue repair, and skin rejuvenation. The author highlights the potential applications of PN in aesthetic treatments, including skin rejuvenation, hair growth promotion, and scar treatment. PN are able to stimulate cellular responses, such as collagen synthesis, angiogenesis, and anti-inflammatory responses, which can improve skin texture, reduce wrinkles, and enhance skin firmness. The article also discusses the mechanisms by which PN exerts its effects, including the activation of signaling pathways, modulation of gene expression, and regulation of cellular metabolism. The author notes that PN can be used alone or in combination with other treatments to enhance their effects (Level V).

Minoretti et al. [31] provide a primer for aesthetic medicine practitioners on clinically actionable topical strategies for addressing the hallmarks of skin aging. The authors focus on the application of polynucleotides, specifically PDRN and PN, in topical treatments for skin rejuvenation. The authors discuss the mechanisms of action of PDRN and PN, which include stimulation of collagen synthesis, improvement of skin elasticity, and enhancement of skin hydration. They highlight the benefits of these polynucleotides in addressing the hallmarks of skin aging, including loss of collagen, elastin, and hyaluronic acid, as well as increased oxidative stress and inflammation. The article reviews the available evidence for the use of PDRN and PN in aesthetic medicine, including studies on their safety and efficacy in improving skin texture, reducing wrinkles, and enhancing facial rejuvenation. The authors conclude that PDRN and PN show promising results in clinical trials, with low risk profiles and minimal adverse effects (Level V).

Yip et al. [32] discuss the current trends and treatments in anti-aging dermatology in Australia. The author highlights the increasing popularity of PN therapy as a non-surgical, non-invasive treatment option for skin rejuvenation. PN therapy involves the injection of PN, which are short chains of nucleotides that stimulate collagen production and improve skin texture. The author notes that PN therapy has gained popularity due to its ability to stimulate collagen production, improve skin elasticity, and reduce fine lines and wrinkles. The article also discusses the use of PN therapy in combination with other treatments, such as botulinum toxin and hyaluronic acid, to enhance its effects. The author suggests that PN therapy is a valuable addition to the armamentarium of anti-aging treatments, offering a safe and effective way to improve skin appearance without surgery or downtime (Level V).

Arora et al. [8] discuss the current trends and advancements in facial fillers for aesthetic purposes. The author highlights the growing popularity of polynucleotides (PNs) as a filler material for facial rejuvenation. The author notes that PNs have gained popularity due to their ability to provide a natural-looking result with minimal downtime and risk of complications. The article discusses the benefits of PNs, including their ability to stimulate collagen production, improve skin elasticity, and provide a natural-looking result with minimal downtime. The author also highlights the importance of proper patient selection, injection technique, and post-treatment care to ensure optimal results (Level V).

Cheng et al. [34] provide an overview of recent advancements in skin fillers, including PN. The authors highlight the growing interest in using PN as a filler material for facial rejuvenation due to its ability to stimulate collagen production and improve skin elasticity. The article discusses the benefits of PN, including their ability to stimulate collagen production and improve skin elasticity, their biodegradable and biocompatible nature, non-animal-derived origin, and potential for use in various facial areas, including nasolabial folds, marionette lines, and lips. The authors also mention the advantages of PN over traditional fillers, such as hyaluronic acid and collagen, including their longer-lasting results and reduced risk of complications (Level V).

## 3. Polynucleotides for Wrinkle Treatment

PN have been extensively explored for their potential in wrinkle treatment, demonstrating promising results in enhancing skin elasticity, hydration, and overall appearance, as detailed in various studies focusing on different application methods and formulations (Table 2).

Lee et al. [36] compare the effects of PN and hyaluronic acid fillers on periocular rejuvenation. Periocular rejuvenation refers to the treatment of the skin around the eyes, which is a common area of aesthetic concern. The study aimed to investigate the safety and efficacy of PN filler compared to hyaluronic acid filler in the periorbital area. The study was a randomized, double-blind, split-face trial where 30 participants were randomly assigned to receive either PN or hyaluronic acid filler in one half of their face, while the other half served as a control. The fillers were injected into the periocular area using a standardized technique. Participants were evaluated at baseline and after 6 months using standardized skin analysis methods, including wrinkle depth, skin elasticity, and skin hydration. The results showed that both fillers improved skin elasticity and hydration, but the PN filler had a greater effect on reducing wrinkle depth and improving skin texture. The study also found that both fillers were well-tolerated, with minimal adverse reactions reported (Level IIb).

Cavallini et al. [37] present a consensus report on the use of PN-HPT (Polynucleotides Highly Purified Technology) in aesthetic medicine. PN-HPT is a type of PN therapy that is claimed to have anti-inflammatory and antioxidant effects. The report was developed by the PN HPT Priming Board, a panel of experts from the Italian College of Aesthetic Medicine Scientific Societies (SIME, AGORÀ, and SIES). The report provides guidelines for the use of PN-HPT in aesthetic medicine, including its indications, contraindications, and recommended dosing and administration methods. The authors highlight the potential benefits of PN-HPT, including its ability to improve skin texture, reduce inflammation, and enhance collagen production. They also discuss potential side effects and adverse reactions, as well as potential interactions with other treatments. The report is based on a critical analysis of existing literature and expert consensus. The authors acknowledge that while there is limited randomized controlled trial data available on PN-HPT, the available evidence suggests that it is safe and effective for aesthetic use. However, they emphasize that more research is needed to fully understand its mechanisms of action and optimal use (Level V).

Pak et al. [38] compare the durability, efficacy, and safety of PN filler with hyaluronic acid (HA) filler for correcting crow’s foot wrinkles. A phase III, randomized, double-blind, matched-pairs, active-controlled trial was conducted with 120 patients, who were randomly assigned to receive either PN or HA filler. The study found that both fillers improved wrinkle correction and skin elasticity at 6 months, but PN filler showed significantly longer duration of action and better efficacy compared to HA filler at 12 months. The study also reported that both fillers were well-tolerated, with no serious adverse effects observed (Level Ib).

Jeong et al. [39] compare the efficacy and safety of two dermal fillers, polycaprolactone (PCL) and PN, for correcting lateral canthal lines. A split-face study design was used, where 20 patients received injections of either PCL or PN on one side of their face, while the other side served as a control. The study aimed to evaluate the safety and efficacy of PN as a new biostimulatory filler for lateral canthal lines. The results showed that both fillers improved facial wrinkles and lines at 12 weeks post-treatment, with similar improvements observed in both groups. However, PN demonstrated a faster onset of action and maintained its effects longer than PCL. Additionally, PN had a lower incidence of adverse reactions, such as swelling and bruising, compared to PCL (Level IIa).

Webb et al. [40] aim to evaluate the role of PN in regenerative and aesthetic medicine. PNs are a class of biomolecules that have been shown to have potential therapeutic benefits in various medical applications. The authors conducted a comprehensive search of major databases and identified 35 studies that met their inclusion criteria. The review found that PN has been used in various clinical settings, including wound healing, tissue engineering, and aesthetic medicine. In these applications, PN has been shown to promote cellular proliferation, migration, and differentiation, as well as improve tissue regeneration and scarless healing. The authors also identified several mechanisms by which PN may exert their effects, including the stimulation of growth factors, modulation of the immune response, and promotion of stem cell activation (Level Ia).

Kim et al. [41] investigate the use of PN for the correction of lateral canthal lines, a common sign of facial aging. The authors used an Antera three-dimensional camera to assess the effects of PN injections on lateral canthal lines in 20 patients. The patients received a single treatment with PN and were evaluated at 1, 3, and 6 months post-treatment. The results showed significant improvements in crow’s appearance at all time points, with a reduction in wrinkle depth and improved skin elasticity. The authors also found that PN treatment was well-tolerated, with no serious adverse events reported (Level IIb).

Oh et al. [42] compare the safety and efficacy of a novel hyaluronic acid-polynucleotide/poly-L-lactic acid composite dermal filler (HPPLA) with other fillers. The study included 30 patients who received HPPLA injections and 30 patients who received other fillers (hyaluronic acid or poly-L-lactic acid). The results showed that HPPLA had higher satisfaction rates and better clinical outcomes compared to the other fillers. Specifically, HPPLA demonstrated improved wrinkle correction, skin elasticity, and patient satisfaction at 6 months post-treatment. The study also reported that HPPLA had a lower rate of adverse effects, such as swelling and bruising, compared to the other fillers. However, both groups experienced similar rates of delayed hypersensitivity reactions (Level IIc).

Lim et al. [43] provide an overview of the use of PN for Asian skin regeneration and rejuvenation. The authors review the current literature on the use of PN, specifically HPT (hyaluronic acid-polyadenylate-polycytidylic acid), in treating various skin concerns in Asian individuals. The article highlights the unique characteristics of Asian skin, including its thinner epidermis and increased collagen production, which can lead to premature aging. The authors discuss the potential benefits of HPT in addressing these concerns, including its ability to stimulate collagen production, improve skin elasticity, and reduce fine lines and wrinkles. The authors also review the current evidence on the safety and efficacy of HPT in various clinical studies, including studies on facial rejuvenation, scar treatment, and skin tightening. They conclude that HPT shows promise in improving skin texture, reducing wrinkle depth, and enhancing overall facial appearance (Level V).

Park et al. [23] report on the use of long-chain PN fillers for skin rejuvenation in five patients. The authors aimed to evaluate the efficacy and safety of this treatment modality for improving facial wrinkles, fine lines, and skin texture. The study involved five patients who received injections of long-chain PN in various areas of their faces. The treatments were performed at intervals of 3–6 months, and the patients were evaluated at each session using standardized photographic analysis and patient satisfaction surveys. The results showed significant improvements in facial wrinkles, fine lines, and skin texture in all five patients. The authors reported that the treatment was well-tolerated, with minimal adverse reactions observed. They also noted that the fillers showed long-lasting effects, with improvements maintained for up to 12 months after treatment (Level IV). One limitation of the study was its study panel of only five subjects, which may limit the statistical power needed for definitive conclusions. Nonetheless, despite this constraint, the study provides valuable preliminary insights into the efficacy and safety of long-chain PN fillers for skin rejuvenation. This exploratory approach lays the groundwork for future research with larger sample sizes to further explore these findings.

## 4. Polynucleotides for Facial Erythema and Scars

PN has shown promising potential in treating facial erythema and scars, with various studies investigating its effectiveness and safety in these applications (Table 3).

Lee et al. [44] investigate the current practices and perceived effectiveness of PN in treating facial erythema among aesthetic physicians. Facial erythema is a common condition characterized by redness or flushing of the skin, often caused by various factors such as rosacea, eczema, or photoaging. PNs are a type of DNA fragment that can be used to regulate gene expression and have been suggested as a potential treatment for facial erythema. The study conducted a survey among 100 aesthetic physicians to gather information on their current practices and opinions regarding the use of PN for treating facial erythema. The results showed that 64% of the physicians reported using PN as part of their treatment regimen, with the majority using it in combination with other therapies such as phototherapy or topical creams. The most common PNs used were salmon sperm DNA and human placental DNA. The physicians’ perceived effectiveness of PN was also investigated. Most physicians (71%) reported that PN was effective in improving skin redness and inflammation, while 44% reported improved patient satisfaction. However, only 26% reported that PN was effective in preventing future episodes of facial erythema (Level IV).

Araco et al. [8] report on a preliminary prospective and randomized study comparing the effectiveness of highly purified PN versus placebo in treating moderate to severe acne scars. The study aimed to evaluate the safety and efficacy of PN treatment for improving the appearance of acne scars. A total of 30 patients with moderate-to-severe acne scars were randomly assigned to receive either PN or placebo injections. The treatments were administered over a period of six months, with patients evaluated at baseline, 3 months, and 6 months using standardized skin analysis methods, including photography and patient satisfaction surveys. The results showed significant improvements in acne scar appearance in both groups, with the PN group demonstrating greater improvements in scar elevation, skin texture, and patient satisfaction. The study also found that the treatment was well-tolerated, with minimal adverse reactions reported (Level IIb).

Palmieri et al. [45] present a real-world insight into the treatment of striae distensae (stretch marks) using an innovative intradermal medical device. The authors report on a case series of 20 patients with striae distensae who received treatment with the device, which combines polyphosphoric acid PN-HPT, hyaluronic acid, and mannitol. The device was applied intradermally to the affected areas, and patients were followed up for 6 months. The results showed significant improvements in the appearance of the stretch marks, with 85% of patients experiencing a noticeable reduction in their severity. Additionally, the treatment was well-tolerated, with only minor side effects reported. The authors suggest that the combination of PN HPT, hyaluronic acid, and mannitol may help to improve the appearance of striae distensae by stimulating collagen production, improving skin elasticity, and reducing inflammation (Level IV).

Kim et al. [46] investigate the preventive effect of PN on post-thyroidectomy scars. A total of 60 patients undergoing thyroidectomy were randomly assigned to two groups: a treatment group that received PN injections around the surgical site and a control group that received saline injections. The study found that the PN group had significantly improved scar quality and reduced scar width compared to the control group at 6 months post-surgery. Additionally, the PN group had a higher rate of excellent or good scar ratings compared to the control group. The study also reported that the PN treatment was well-tolerated, with no serious adverse effects observed (Level Ib).

## 5. Polynucleotides for Vulvar and Vaginal Rejuvenation

PN has shown promising results in the rejuvenation of vulvar and vaginal tissues, offering significant improvements in symptoms such as vaginal dryness, itching, and atrophy among postmenopausal women (Table 4).

Palmieri et al. [47] report on the use of biorevitalization with PN and hyaluronic acid (HA) for treating postmenopausal labia majora. The authors aimed to evaluate the efficacy and safety of this treatment modality in improving vaginal dryness, itching, and atrophy. The study involved 20 postmenopausal women who received injections of PN and HA in their labia majora. The treatments were performed at intervals of 3–6 months, and the patients were evaluated at each session using standardized questionnaires and physical examinations. The results showed significant improvements in vaginal dryness, itching, and atrophy in all patients. The authors reported that the treatments were well-tolerated, with minimal adverse reactions observed. They also noted that the patients experienced increased vaginal lubrication and reduced symptoms of menopause-related vulvovaginal atrophy (Level IV).

Palmieri et al. [22] explore the effects of vulvar rejuvenation with PN-HPT on postmenopausal sexual life disruption. A retrospective analysis was conducted on 20 postmenopausal women who underwent vulvar rejuvenation with PN-HPT. The study found that all patients experienced improvements in vulvar elasticity, skin hydration, and reduced vaginal dryness after treatment. Additionally, 80% of patients reported improved sexual function, including increased libido, enhanced pleasure, and reduced pain during intercourse. The study also reported that patients experienced no serious adverse effects or complications (Level IV).

Angelucci et al. [48] investigate the efficacy of intradermal hyaluronic acid plus polynucleotides (Hyal/PN) in treating vulvovaginal atrophy (VVA). The pilot study included 15 postmenopausal women with VVA who received a single injection of Hyal/PN into the vulvar tissue. The results showed significant improvements in symptoms of VVA, including vaginal dryness, itching, and dyspareunia, at 6 months after treatment. The study also found significant increases in vaginal pH and vaginal mucosa thickness, indicating improved hydration and elasticity. Additionally, 86% of patients reported improved sexual function and satisfaction (Level IV).

## 6. Polynucleotides for Miscellaneous Applications

PN has shown promising potential in various miscellaneous applications in aesthetic and dermatological treatments, ranging from facial rejuvenation and wrinkle treatment to the management of infra-orbital dark circles and melasma, as evidenced by several studies (Table 5).

Park et al. [49] discuss the treatment of infra-orbital dark circles, a common aesthetic concern. The authors focus on the use of PN as a treatment option for this condition. The authors review various etiologies of infra-orbital dark circles, including vascular, fat pad, and skin-related causes. They highlight the limitations of current treatments, such as hyaluronic acid fillers and laser therapy, and suggest that PN may offer a promising alternative. PNs are biodegradable polymers that can be used to stimulate collagen production, improve skin elasticity, and reduce the appearance of fine lines and wrinkles. The authors suggest that PN can be used to treat infra-orbital dark circles by improving the overall appearance of the under-eye area. The authors review several studies that have used PN to treat various skin concerns, including facial wrinkles and scars. They also discuss the potential benefits of using PN in combination with other treatments, such as platelet-rich plasma (PRP) therapy (Level IIIa).

Yogya et al. [50] investigate the efficacy and safety of using non-insulated microneedle radiofrequency (MRF) alone or in combination with polynucleotides for treating periorbital wrinkles. A total of 120 participants were randomly assigned to three groups: MRF alone, MRF with polynucleotides, and a control group that received no treatment. The study found that both MRF alone and the combination therapy resulted in significant improvements in wrinkle depth and skin elasticity compared to the control group at 6 months post-treatment. However, the combination therapy showed greater improvements in wrinkle depth and skin elasticity compared to MRF alone. The study also found that both treatments were well-tolerated, with minimal adverse effects reported. The most common side effects were mild redness and swelling, which resolved within a few days (Level IIa).

Gulfan et al. [51] investigate the efficacy and safety of using non-insulated microneedle radiofrequency (MNRF) alone or in combination with polynucleotides for the treatment of melasma. A pilot study was conducted with 30 patients, who were randomly assigned to receive either MNRF alone or MNRF with polynucleotides. The study found that both treatments improved melasma severity and skin elasticity at 6 months, but the combination treatment showed better efficacy and faster improvement. The study reported that both treatments were well-tolerated, with no serious adverse effects observed (Level IIb).

## 7. Conclusions

The skin, the body’s largest organ, maintains homeostasis through complex neuroendocrine and immune interactions, producing various hormones like melanotropins and vitamin D3 locally to respond to stress, and its disturbance can lead to skin and systemic pathologies [52,53].

Photoaging and photocarcinogenesis, caused by solar ultraviolet radiation (UVR), result in DNA damage, oxidative stress, immunosuppression, and extracellular matrix remodeling [54]. UVR is primarily known for its detrimental effects, such as cancerogenesis, skin aging, eye damage, and autoimmune disorders. However, beyond its role in vitamin D3 production, UVR also contributes to maintaining homeostasis through complex local and systemic responses. These responses, triggered by UVR exposure, involve chemical, hormonal, immune, and neural signals coordinated by the cutaneous neuro-immuno-endocrine system, which help regulate overall body homeostasis. The skin, equipped with sensory and computing capabilities, communicates with the central nervous, endocrine, and immune systems to counteract environmental stressors and maintain balance. While UVR can induce immunosuppression and activate brain and endocrine centers, it also has therapeutic potential for conditions like addiction, mood disorders, autoimmune diseases, and neurodegenerative conditions. Additionally, vitamin D and melatonin derivatives play crucial roles in these processes, with vitamin D3 being synthesized in response to UVB exposure and influencing bone health, immune function, and mood, while melatonin derivatives, regulated by UVR, impact circadian rhythms and antioxidant defense [55,56,57].

PN plays a significant role in mitigating photoaging by promoting tissue repair and regeneration, thereby improving skin texture, reducing wrinkles, and enhancing overall skin appearance. Understanding these mechanisms can lead to novel treatments that harness the beneficial aspects of UVR while mitigating its harmful effects. 

PN have increasingly gained prominence in aesthetic and cosmetic applications globally due to their high biocompatibility and natural origin from chum salmon or trout gonads, which sets them apart from other biostimulators that are typically synthetic polymer-based [11,17,58,59,60,61,62,63].

**Figure 3:**
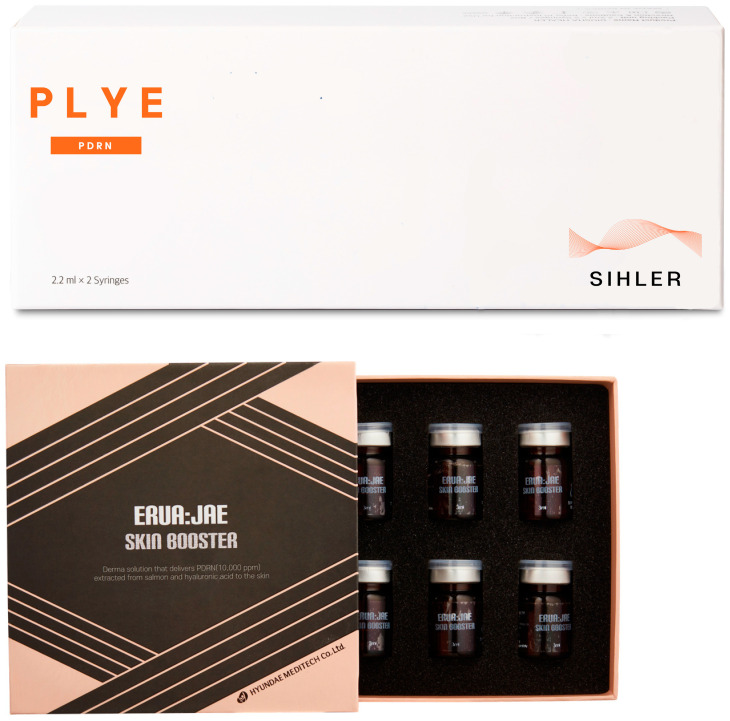
Polydeoxyribonucleotide (PDRN) products are now utilized for aesthetic purposes. The image above shows a pre-syringed PDRN product named PLYE (Sihler Inc., Seoul, Republic of Korea), while the image below features a vial type of PDRN called Eruajae (Hyundae Meditech Co., Wonju, Republic of Korea).

PN are distinct from polydeoxyribonucleotides (PDRN) in several key aspects: 1. PN are derived from testes, whereas PDRN are sourced from sperm cells; 2. PN possess longer nucleotide chains; 3. PN exhibit a higher molecular weight; and 4. PN have a scaffold structure that is not present in PDRN (Figure 3) [64,65].

One unique finding is that PDRN promoted cyclobutene pyrimidine dimer (CPD) repair in UVB-exposed dermal fibroblasts [10]. Recent discoveries showed PDRN and its properties in anti-melanogenesis [14,66], anti-allodynic [67], mitochondrial biogenesis [14], and even fat browning for potential anti-obesity applications [68]. On the other hand, polynucleotides are longer chains of nucleotides, which have a much less robust number of research articles.

It is important to note that in all of the above studies, (1) there were no serious adverse events reported (safety) and (2) there is no objective data on the mechanism of action. It is hypothesized that, due to the similarity of PN and PDRN molecular structures, PN will have a mechanism of action similar to PDRN. However, up to date, this has not been scientifically demonstrated [58,59,60].

The shape of PN images reveals that the majority of PN “cells” or “shapes” are five-sided pentagons and six-sided hexagons, with slight variations in size and shape due to the gel-like consistency of the product. This contrasts with other biostimulator polymers, which typically have spherical or irregular particles. Hexagonal shapes are common in nature, seen in structures like bubbles, diamonds, graphite, snowflakes, muscle fibers, and honeycombs, due to their energetically efficient configurations. In living tissues, hexagonal arrangements balance internal and external tensional forces. Studies have shown that hexagonal cells in scaffolds offer superior porosity and mechanical strength compared to triangular or square cells, significantly influencing scaffold performance at both microscopic and macroscopic levels [69,70,71,72,73,74,75].

The size of the length of the cell walls ranges from approximately 1–7 µm, with most falling within the 4–6 µm range, while the diameter of the cells is about 3–5 µm. The PN scaffold structure is significantly smaller than the average fibroblast, which has a diameter of approximately 28 µm, potentially allowing more fibroblast binding to the scaffold walls. According to a model proposed in 1987, the number and density of receptor adhesive sites between the cell (integrin binding sites) and substrate (ligand binding sites) influence fibroblast activity. It remains to be determined if the PN scaffold size is optimal for fibroblast binding. Research indicates that in older skin, extracellular matrix (ECM) collagen becomes more fragmented and shorter, reducing attachment points for fibroblasts, leading to cell shrinkage, loss of mechanical stimulation, and decreased collagen production. Injection of PN could potentially enhance the matrix scaffold in the ECM, facilitating fibroblast attachment and functionality. Studies have shown that small, rounded fibroblasts placed in constrained matrices display a spread morphology and restore their mechanical properties [76,77,78,79,80].

The SEM images of PN reveal that its cells are intricately connected and arranged in a tessellated pattern without gaps, resembling a hexagonal structure due to its efficient perimeter-to-area ratio. While the cell walls are not of equal length, the 3-D perspective of PN must be considered to understand its in vivo effects. The Wearie–Phelan structure, composed of pentagons and hexagons, offers an efficient model for space tessellation with equal volume cells and minimal surface area. PN’s pentagonal and hexagonal cell shapes observed in SEM images closely resemble this structure, suggesting potential insights into its mechanical properties and role in the extracellular matrix. The polyhedral tessellation structure of PN, with its organized and interconnected nature, reflects biological architecture and may have applications in tissue regeneration, stem cell research, bioengineering, and as a delivery vehicle [81].

In 2009, it was demonstrated that DNA can be used to build nano-structures with precise control. Further research in 2023 showed that DNA molecules can serve as nano-lattices due to their superior strength-to-weight ratios. When combined with materials like silica, they form a lightweight and high-strength framework, enabling the production of diverse lattice types and leading to varied mechanical responses in material development. Additionally, DNA crystal films formed through self-assembly have potential applications in UV radiation protection and wound healing when applied to the skin [82,83,84].

PN, due to their ability to modulate the elasticity of the extracellular matrix (ECM) via their scaffold structure, show promise for future stem cell research and applications, both in vivo and in vitro. Preconditioning stem cells with specific in vitro matrix conditions can help direct lineage specification, potentially overcoming unsuitable in vivo environments. The stiffness of a substrate has been shown to influence cell response and differentiation, and the alignment and size of nanogratings can direct stem cell differentiation without biomechanical factors. These findings highlight the critical role of ECM composition in guiding stem cell differentiation [13,85,86].

PN presents unique characteristics, such as thin walls and polyhedral shapes, that offer potential for innovative delivery mechanisms, including controlled and sustained release of encapsulated substances, essential for advanced drug delivery systems [13,15,87]. Their applications extend to both oral and injectable drug delivery, where precise dosage control is critical [87]. PN’s scaffold structure has been used effectively in bone tissue repair and regeneration, demonstrating promising results. The ideal scaffold should possess low antigenicity, promote cell adhesion and proliferation, have high mechanical strength, and feature an interconnected porous architecture for uniform cell distribution and tissue formation [88,89]. Scaffolds must mimic the nanofibrous collagen ECM, supporting cell ingrowth and ensuring efficient transport of growth factors, oxygen, nutrients, and waste products while allowing for adequate vascularization to prevent necrosis. Additionally, scaffolds should biodegrade at a rate proportional to new tissue formation, exit the body without interfering with other tissues, and exhibit high biocompatibility with minimal immune response. The structural pattern of PN can be applied to biotensegrity principles, which emphasize the role of continuous tension and compression elements in maintaining structural stability and regulating cellular biochemical responses through mechanotransduction [90,91,92,93,94,95,96,97,98,99].

**Figure 4:**
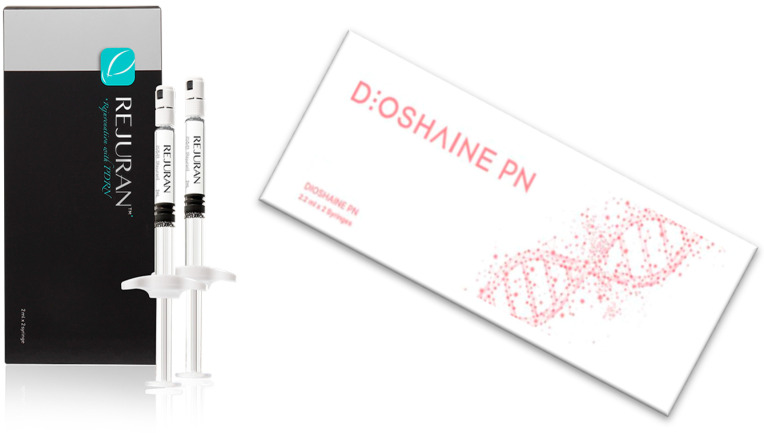
PN has increasingly gained prominence in aesthetics and cosmetic applications. Numerous PN products are available in the market for aesthetic purposes, with notable examples including Rejuran (Pharmaresearch Inc., Gangneung-si, Republic of Korea) and Doshaine PN (Hyundae Meditech Co., Wonju, Republic of Korea), both of which are offered in prefilled syringes.

While there is limited high-quality evidence available on the use of polynucleotides in aesthetic medicine, the existing literature suggests that they may be a promising treatment option for various aesthetic conditions (Figure 4). 

Further research is needed to confirm their efficacy and optimal use in different indications. As the field continues to evolve, it is essential to stay up-to-date with the latest research and developments to provide evidence-based treatments for our patients. High-quality randomized controlled trials are necessary to establish the effectiveness of polynucleotides compared to other treatments. Further studies are needed to investigate the optimal dosing and administration methods for polynucleotides [2,22,23,24,29,36,42,100].

The review has limitations due to a scarcity of high-quality, large-scale randomized controlled trials (RCTs) on the efficacy and safety of polynucleotides in aesthetic applications. Most evidence is derived from observational studies, case reports, and small pilot studies, which may not provide robust or generalizable conclusions. Additionally, there is considerable variability in study designs, patient populations, treatment protocols, and outcome measures across the reviewed studies. This heterogeneity makes it challenging to compare results directly and draw definitive conclusions about the overall effectiveness of polynucleotides. Furthermore, many of the studies included in the review have relatively short follow-up periods. The long-term efficacy and safety of polynucleotide treatments remain uncertain, and further research with extended follow-up is necessary to evaluate the sustainability of treatment outcomes. The literature review has highlighted the potential of polynucleotides in aesthetic medicine, with a focus on their use in various aesthetic conditions. However, the studies reviewed suggest that polynucleotides may be effective in improving skin texture, reducing wrinkle depth, and enhancing facial appearance. While the existing evidence is promising, further research is needed to confirm their efficacy and optimal use in different indications. High-quality randomized controlled trials are necessary to establish the effectiveness of polynucleotides compared to other treatments. Additionally, more research is required to investigate the optimal dosing and administration methods for polynucleotides. As the field continues to evolve, it is essential for clinicians to stay up-to-date with the latest research and developments to provide evidence-based treatments for their patients. Further research is needed to confirm the efficacy and optimal use of polynucleotides in different indications.

## Figures and Tables

**Table 1:** Polynucleotide for General Skin Condition.

Study	Category	Summary	Evidence Level
Yi et al. [26]	Skin Boosters	Overview of injectable treatments to improve skin texture and appearance, focusing on PN.	V
Morganti et al. [27]	Delivery Systems for Cosmeceuticals	Explores the use of PN as a novel delivery system for cosmeceuticals.	IIa
Yi et al. [28]	Skin Boosters	Reviews various types of skin boosters used in aesthetic treatments, focusing on PN.	Ia
Lis et al. [29]	Treatment for Neck Skin Aging	Investigates the use of PN for reducing signs of aging in neck skin.	IV
Cesare et al. [30]	Biorevitalization Protocols	Describes a combined treatment protocol for biorevitalization, including PN.	IV
Samizadeh et al. [7]	General Overview of PN in Aesthetic Medicine	Provides an overview of the role of PN in aesthetic medicine.	V
Minoretti et al. [31]	Topical Strategies for Skin Aging	Provides a primer for aesthetic medicine practitioners on topical strategies for skin rejuvenation using PN.	V
Yip et al. [32]	Trends in Anti-Aging Dermatology	Discusses current trends and treatments in anti-aging dermatology, focusing on PN therapy.	V
Arora et al. [33]	Facial Fillers	Discusses advancements in facial fillers for aesthetic purposes, highlighting PN.	V
Cheng et al. [34]	Facial Fillers	Provides an overview of recent advancements in skin fillers, including PN.	V

**Table 2:** Polynucleotides for Wrinkle Treatment.

Study	Focus	Methodology	Key Findings	Level
Lee et al. [36]	Periocular rejuvenation	Randomized, double-blind, split-face trial with 30 participants	PN filler improved skin elasticity and texture more than hyaluronic acid filler; minimal adverse reactions	IIb
Cavallini et al. [37]	PN-HPT in aesthetic medicine	Consensus report by expert panel	PN-HPT improves skin texture, reduces inflammation, and enhances collagen production; safe and effective	V
Pak et al. [38]	PN vs. hyaluronic acid filler for crow’s feet	Phase III, randomized, double-blind trial with 120 patients	PN filler had longer duration of action and better efficacy than HA filler; well-tolerated	Ib
Jeong et al. [39]	PN vs. polycaprolactone (PCL) for canthal lines	Split-face study with 20 patients	Both fillers improved wrinkles; PN had faster action and fewer adverse reactions	IIa
Webb et al. [40]	Role of PN in regenerative and aesthetic medicine	Comprehensive review of 35 studies	PN promotes cellular proliferation, migration, and differentiation; beneficial for tissue regeneration	Ia
Kim et al. [41]	PN for lateral canthal lines	Study with 20 patients	Significant improvement in wrinkle appearance; well-tolerated	IIb
Oh et al. [42]	HPPLA vs. other fillers	Study with 30 patients	HPPLA had higher satisfaction and better outcomes; lower adverse effects	IIc
Lim et al. [43]	PN for Asian skin regeneration	Literature review	HPT stimulates collagen production and improves skin elasticity; effective for facial rejuvenation	V
Park et al. [23]	Long-chain PN fillers for skin rejuvenation	Study with 5 patients	Significant improvements in wrinkles and texture; long-lasting effects	IV

**Table 3:** Polynucleotides for Facial Erythema and Scars.

Study	Focus	Methodology	Results	Evidence Level
Lee et al. [44]	Current practices and perceived effectiveness of PN in treating facial erythema	Survey of 100 aesthetic physicians; evaluated use and effectiveness of PN for facial erythema	64% used PN; 71% reported effectiveness in reducing redness and inflammation; 44% reported improved patient satisfaction; 26% in preventing future episodes.	Level IV
Araco et al. [8]	Comparing effectiveness of highly purified PN vs. placebo in treating moderate to severe acne scars	Randomized study with 30 patients; PN or placebo injections; evaluations at baseline, 3, and 6 months	Significant improvements in scar appearance, skin texture, and patient satisfaction in the PN group; well-tolerated with minimal adverse reactions.	Level IIb
Palmieri et al. [45]	Treatment of striae distensae (stretch marks) using an innovative intradermal medical device	Case series with 20 patients; device combining polyphosphoric acid (PN HPT), hyaluronic acid, and mannitol applied intradermally	85% of patients showed noticeable reduction in stretch mark severity; improved collagen production, skin elasticity, and reduced inflammation; well-tolerated.	Level IV
Kim et al. [46]	Preventive effect of PN on post-thyroidectomy scars	Randomized study with 60 patients; PN injections around surgical site vs. saline injections; evaluations at 6 months	PN group showed significantly improved scar quality, reduced scar width, and higher rate of excellent/good scar ratings; well-tolerated with no serious adverse effects.	Level Ib

**Table 4:** Polynucleotides for Vulvar and Vaginal Rejuvenation.

Study	Purpose/Objective	Study Design	Sample Size	Treatment	Outcomes	Level of Evidence
Palmieri et al. [47]	Evaluate efficacy and safety of PN and HA for treating postmenopausal labia majora	Prospective observational study	20	PN and HA injections in labia majora	Significant improvements in vaginal dryness, itching, and atrophy; minimal adverse reactions	IV
Palmieri et al. [22]	Effects of PN-HPT on postmenopausal sexual life disruption	Retrospective analysis	20	Vulvar rejuvenation with PN-HPT	Improvements in vulvar elasticity, skin hydration, vaginal dryness; 80% reported improved sexual function	IV
Angelucci et al. [48]	Efficacy of Hyal/PN in treating vulvovaginal atrophy (VVA)	Pilot study	15	Single injection of Hyal/PN	Improvements in VVA symptoms (vaginal dryness, itching, dyspareunia), increased vaginal pH and mucosa thickness, 86% reported improved sexual function and satisfaction	IV

**Table 5:** Summarized table of Polynucleotides for Miscellaneous Applications.

Study	Application	Key Findings	Level
Park et al. [49]	Infra-orbital Dark Circles	PN is used to treat dark circles by improving skin elasticity and reducing fine lines and wrinkles.	IIIa
Yogya et al. [50]	Periorbital Wrinkles	Non-insulated microneedle radiofrequency (MRF) with PN showed greater improvements in wrinkle depth and skin elasticity compared to MRF alone.	IIa
Gulfan et al. [51]	Melasma	Non-insulated microneedle radiofrequency (MNRF) with PN showed better efficacy and faster improvement in melasma severity and skin elasticity compared to MNRF alone.	IIb
